# Dapsone as a Current Option for the Treatment of Autoimmune Bullous Diseases with Autoimmunity to Non-Enzymes: A Retrospective Study from a Single Central European Referral Center

**DOI:** 10.3390/medicina60081324

**Published:** 2024-08-15

**Authors:** Maciej Marek Spałek, Magdalena Jałowska, Natalia Welc, Monika Bowszyc-Dmochowska, Marian Dmochowski

**Affiliations:** 1 Autoimmune Blistering Dermatoses Section, Department of Dermatology, Poznan University of Medical Sciences, 60-355 Poznan, Poland; mspalek@ump.edu.pl (M.M.S.); mjalowska@ump.edu.pl (M.J.); natalia.welc@usk.poznan.pl (N.W.); mdmochowski@ump.edu.pl (M.D.); 2 Cutaneous Histopathology and Immunopathology Section, Department of Dermatology, Poznan University of Medical Sciences, 60-355 Poznan, Poland

**Keywords:** autoimmune bullous diseases, dapsone, glucocorticosteroids

## Abstract

*Background and Objectives*: Dapsone (DP) is employed in the management of various skin conditions, including autoimmune bullous diseases to non-enzymes (n-eAIBDs). This study aimed to assess the advantages and safety profile of DP treatment in n-eAIBDs patients. The evaluation focused on clinical remission, reduction in glucocorticosteroid (GCS) usage, and adverse incidents during a 12-month observation in a dermatology department at a Central European university. *Materials and Methods*: Our retrospective study included forty-one patients who met the inclusion criteria, comprising nineteen with pemphigus vulgaris, nine with pemphigus foliaceus, four with bullous pemphigoid, and nine with mucous membrane pemphigoid, including one patient with Brunsting–Perry pemphigoid. Patients received 25–50 mg/day of DP along with oral GCSs for a year, with a subsequent dose reduction where feasible. *Results*: The mean decreases in prednisone-equivalent dosages across all groups after 2, 6, and 12 months of DP treatment were 45.66%, 65.77%, and 63.03%, respectively. Throughout the 12-month observation period, 21.62% of patients experienced a relapse, while the remaining patients attained either complete or partial remission with minimal therapy. Adverse incidents were observed in 29.27% of patients; these were mild or moderate, and no severe negative effects were observed. *Conclusions*: DP is an effective and affordable choice to support the treatment of n-eAIBDs, but it may not be sufficient for long-term management in certain patients with severe n-eAIBDs.

## 1. Introduction

Autoimmune bullous diseases comprise a heterogeneous group of disorders caused by the presence of antibodies specific to structural proteins or enzymes expressed in the skin and mucous membranes [1]. The autoimmune process leads to the development of vesicles, blisters, and eventually erosions. In intraepithelial diseases, particularly the pemphigus group, and subepithelial diseases, specifically the pemphigoid group and epidermolysis bullosa acquisita (EBA), autoimmunity targets structural proteins rather than enzymes, classifying them as non-enzyme-targeting autoimmune blistering diseases (n-eAIBDs). Conversely, in dermatitis herpetiformis (DH), autoimmunity targets enzymes instead of structural proteins [2]. The gold standard in diagnosing n-eAIBDs remains direct immunofluorescence (DIF), which enables a precise diagnosis based on the types of immunoglobulins, their subtypes, and the binding patterns [3]. Moreover, serology is frequently used to detect and assess circulating antibodies, for instance, with an enzyme-linked immunosorbent assay (ELISA) [4].

Not only do n-eAIBDs have a strong, negative influence on the patients’ quality of life but they may also result in a significantly increased morbidity [5,6]. That is why establishing the appropriate therapeutic approach is essential. The first-line medications for n-eAIBDs are still glucocorticosteroids (GCSs), both topical and systemic, which are efficacious in reducing mortality and improving the prognosis in patients. However, they have several side effects, including osteoporosis, diabetes, hypertension, gastrointestinal ulcers, secondary adrenal insufficiency, cataracts, or infections [2]. Steroid-sparing drugs, such as methotrexate (MTX), cyclosporine, azathioprine (AZA), mycophenolate mofetil (MMF), cyclophosphamide, or dapsone (DP), should be introduced as soon as possible to reduce the cumulative dose of GCSs and prevent patients from developing side effects after the GCS therapy [7].

DP (4,4′-diaminodiphenylsulfone) is a sulfone-derived drug primarily used to treat human leprosy [8]. It acts both as an antimicrobial and anti-inflammatory agent. As an antibiotic, it keeps microorganisms dependent on endogenous folic acid synthesis from growing. The anti-inflammatory qualities of DP such as the abilities to withhold neutrophil recruitment, shield cells from neutrophil- and eosinophil-mediated injury by producing oxygen-derived radicals, and diminish prostaglandin and leukotriene release make it an effective substance in the treatment of many dermatological conditions, like acropustulosis infantilis, erythema elevatum diutinum, prurigo pigmentosa, or subcorneal pustular dermatosis (Sneddon–Wilkinson disease) [8,9,10,11].

DP is a GCS-sparing medication in treating n-eAIBDs [2]. Apart from DH and linear IgA bullous dermatosis, for which DP is established as the first-line treatment, larger studies on the efficacy of DP on other n-eAIBDs are needed.

The main aim of this research was to evaluate the effectiveness of DP in managing n-eAIBDs during a 12-month observation period.

## 2. Materials and Methods

This retrospective study examined the medical records of patients diagnosed with n-eAIBDs who received DP therapy at the dermatology department in Poznan between 2014 and 2022. The research utilized the following exclusion criteria:

- Patients taking less than 10 mg/day of prednisolone equivalent prior to their initial DP dose, excluding those with absolute contraindications to GCSs;
- Individuals with fewer than 10 active lesions (including blisters, erosions, or newly developed areas of erythema) before beginning DP therapy;
- Subjects without follow-up data for a minimum of one year during DP treatment;
- Patients who had been treated previously with immunosuppressants, rituximab (RTX), or intravenous immunoglobulin (IVIG);
- Individuals with a history of allergic reactions to the prescribed medication.

During observation, five patients discontinued DP therapy, two of them because of severe gastrointestinal symptoms resistant to symptomatic treatment and three of them due to problems with DP availability. Following the implementation of these exclusion criteria, we qualified 41 patients to participate in the study.

The identification of pemphigus vulgaris (PV), pemphigus foliaceus (PF), bullous pemphigoid (BP), and mucous membrane pemphigoid (MMP) included the assessment of clinical manifestations, histological findings, and immunological tests. These tests encompassed DIF, indirect immunofluorescence, and multiplex ELISA by Euroimmun (Luebeck, Germany) to detect IgG antibodies against DSG-1, DSG-3, BP 180, BP 230, envoplakin, and type VII collagen [12].

All participants in the research received 100 mg DP/day for 2 weeks, and then the dosage was reduced to 25–50 mg/day. We did not determine the level of glucose-6-phosphate dehydrogenase (G6PD) activity in our group of patients since Poland is not a malaria-endemic area [13,14].

Evaluations were performed at 2, 6, and 12 months post the implementation of the first DP dosage. Where it was possible, the dose of GCSs was reduced. All patients underwent measurements of their methemoglobin levels every 3 months.

We utilized the criteria outlined by Murrell DF et al. to determine disease activity management. Quoting the authors of the criteria: “A complete remission off therapy (CRNT) is defined as the absence of new and/or established lesions while the patient is off all systemic therapy for at least 2 months. A complete remission on therapy (CRMT) is defined as the absence of new or established lesions while the patient is receiving minimal therapy. Minimal therapy is defined as less than or equal to 10 mg/d of prednisone (or the equivalent) and/or minimal adjuvant therapy for at least 2 months. Minimal adjuvant therapy is defined as half of the dose required to be defined as treatment failure. A partial remission off therapy (PRNT) is defined as the presence of transient new lesions that heal within 1 week without treatment and while the patient is off all systemic therapy for at least 2 months. A partial remission on minimal therapy (PRMT) is defined as the presence of transient new lesions that heal within 1 week while the patient is receiving minimal therapy, including topical steroids” [15]. 

## 3. Results

Our retrospective research enrolled 41 participants, including 19 with PV, 9 with PF, 4 with BP, and 9 with MMP. The cohort included 19 males and 22 females, with an average age of 62.07 ± 13.43 years (mean ± SD), spanning 33 to 90 years. The mean baseline prednisone-equivalent dosage was 24.86 ± 12.16 mg.

Comprehensive details regarding the general characteristics of each patient group are presented in Table 1.

Among the patients, the most common comorbidities included hypertension, type 2 diabetes, heart failure, chronic obstructive pulmonary disease, and hypothyroidism.

The occurrence of comorbidities in individual patient groups is presented in Table 2.

The mean initial prednisone-equivalent dose in all groups stood at 24.86 ± 12.16 mg, which progressively tapered among patients with controlled or stabilized disease. After the 2 months of administration of DP, the mean prednisone-equivalent dosage was 13.51 ± 6.86 mg. This figure further decreased to 8.51 ± 3.65 mg at 6 months and reached 9.19 ± 7.31 mg at the end of the 12-month period. As such, the dosage reductions were 45.66%, 65.77%, and 63.03% at 2, 6, and 12 months, respectively.

**Figure 1:**
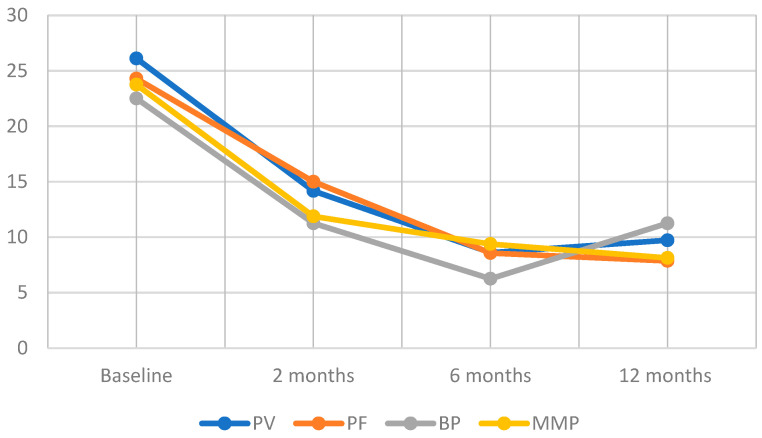
Reduction in average prednisone equivalent dose (mg) over DP treatment in patients divided by the type of n-eAIBDs diagnosis. PF, pemphigus foliaceus; PV, pemphigus vulgaris; MMP, mucous membrane pemphigoid; BP, bullous pemphigoid.

Information regarding the specific patient groups’ reactions to DP therapy over the 1-year follow-up period is illustrated in Figure 1.

After two months of DP therapy, 4.88% of patients experienced disease recurrence, while the remainder achieved partial or complete remission. During the six-month observation period, the rate of disease relapse increased to 9.76%, doubling to 19.51% after 12 months of DP administration. By the end of the observation period, 53.66% of participants had achieved CRMT, and 26.83% had achieved PRMT. None of the patients had CRNT.

**Figure 2:**
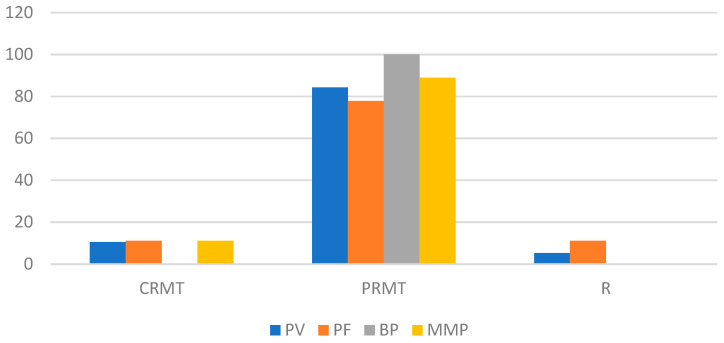
Response to DP therapy (percentage of patients) after 2 months by type of n-eAIBDs diagnosis.

**Figure 3:**
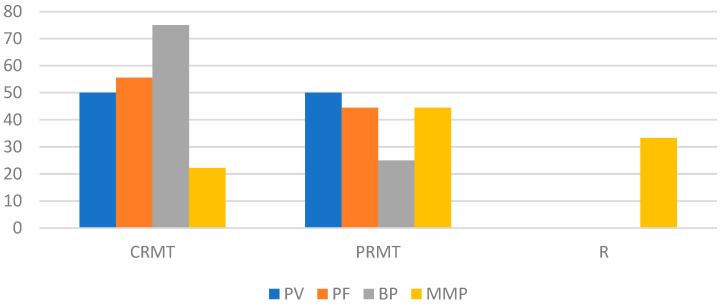
Response to DP therapy (percentage of patients) after 6 months by type of n-eAIBDs diagnosis.

**Figure 4:**
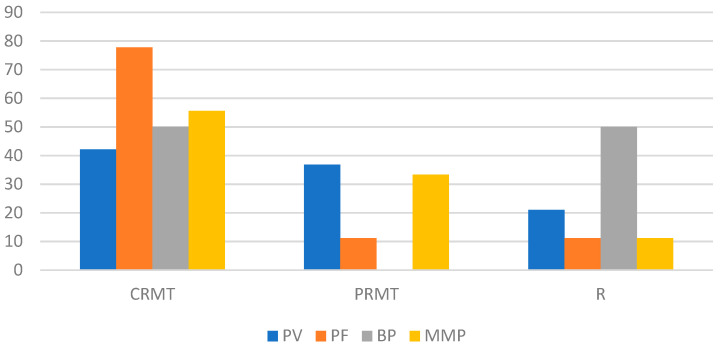
Response to DP therapy (percentage of patients) after 12 months by type of n-eAIBDs diagnosis.

The responses to DP treatment, categorized by n-eAIBDs diagnosis after 2, 6, and 12 months, are depicted in Figure 2, Figure 3 and Figure 4.

In our study, we enrolled patients with various types of n-eAIBDs, all of whom had active disease. Each participant was taking DP, which they obtained from neighboring countries, predominantly Germany. It is noteworthy that acquiring DP in Poland is challenging, as it requires a target import application. This complex administrative process involves obtaining consent from a provincial consultant in dermatology and the Ministry of Health, extending the drug acquisition process to several months and limiting its availability.

**Figure 5:**
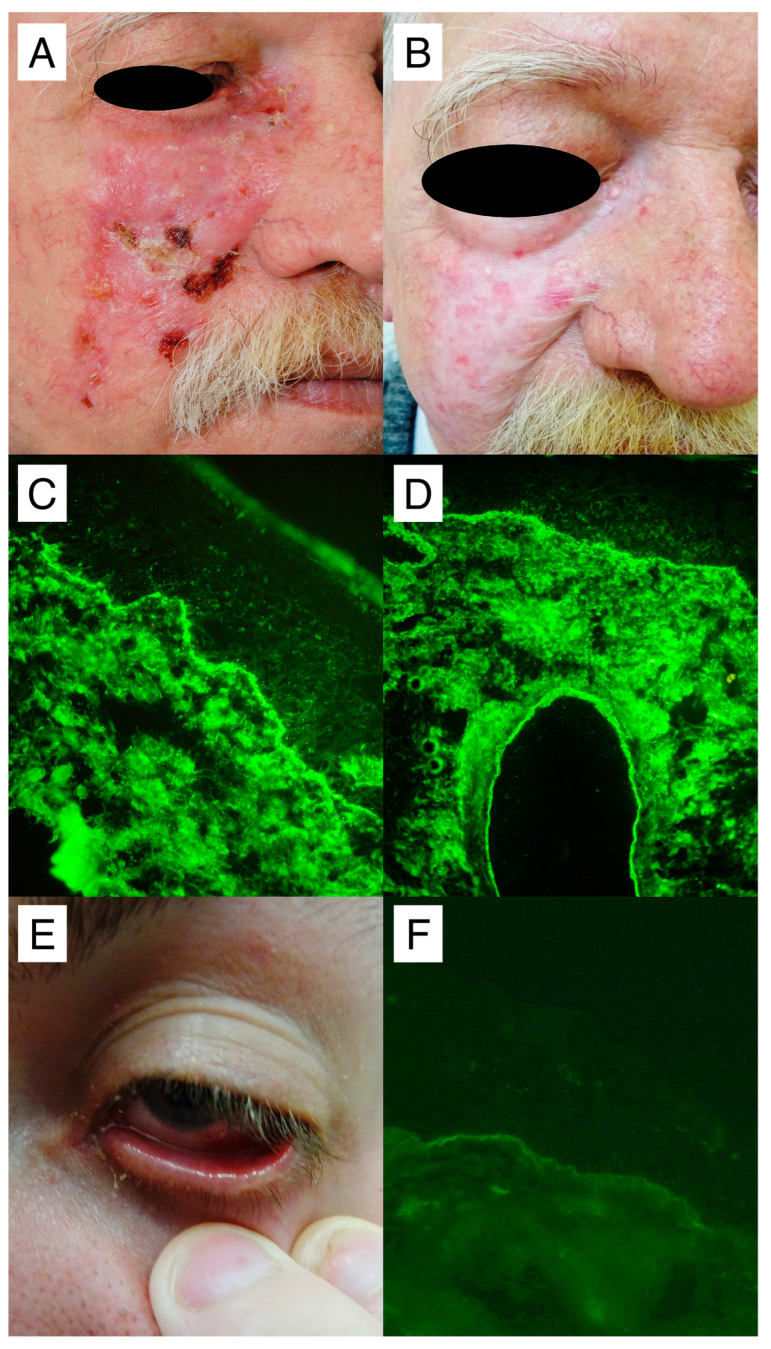
Representative patients with subepithelial autoimmune bullous diseases: A middle-aged male with Brunsting–Perry pemphigoid, who was also being treated for epilepsy with levetiracetam, presented with scarring, flaccid bullae, and erosions, some of which were covered with bloody and yellowish crusts on the right cheek and the medial angle of the eye (**A**). Combined treatment with DP, oral methylprednisolone, and the replacement of levetiracetam with tiagabine brought his disease under control, resulting in only residual scarring, with neither blisters nor erosions visible (**B**). DIF of perilesional skin, observed with short-arc mercury lamp-operated microscopy, revealed IgG4 (++) linear deposits along the basement membrane of extrafollicular (**C**) and follicular (**D**) epithelia. Deposits of C3 (+) with the same pattern, but not IgA, IgG, or IgG1, were also detected. Monoparametric ELISA detected a slightly elevated level of IgG antibodies to BP180 (24.537 RU/mL, cut-off level 20 RU/mL), but not to BP230. A young male with ocular MMP presented with erosions on the bulbar conjunctiva and palpebral conjunctiva of the lower eyelid of the left eye (**E**). DIF of perilesional conjunctiva, observed with blue-light-emitting diode technology-operated microscopy, revealed IgG1 (+) linear deposits along the basement membrane of the conjunctival epithelium (**F**), while a multiplex ELISA gave normal readings. All photomicrographs were taken at the original objective magnification of x40.

Particularly noteworthy are two cases of patients diagnosed with Brunsting–Perry pemphigoid and ocular MMP who were successfully treated with DP, as shown in Figure 5.

Twelve patients experienced side effects, the most common being nausea, reported by eight patients. Additionally, two patients reported skin itching, and two experienced mild headaches, with no instances of methemoglobinemia. Patients who reported adverse reactions were scheduled for more frequent check-ups and, when necessary, were provided with symptomatic medications or had their DP dosage reduced from 50 mg/day to 25 mg/day. Ultimately, all patients resolved their symptoms and continued DP treatment.

## 4. Discussion

This article is the third in a series discussing our clinical laboratory research on non-GCS treatment options for n-eAIBDs at a Central European referral center [16,17].

The potential GCS-sparing effect of DP is attributed to its multifaceted anti-inflammatory properties. One key mechanism is the inhibition of neutrophil chemotaxis and the formation of reactive oxygen species (ROS), both of which are crucial in the pathogenesis of n-eAIBDs [18,19]. Additionally, inflammatory infiltrates in n-eAIBDs may include eosinophils, which contribute to spongiosis in the early phase of pemphigus diseases. Studies indicate that DP inhibits eosinophil myeloperoxidase, an inflammatory mediator in spongiosis, suggesting that early administration of DP could mitigate the initial inflammatory processes in n-eAIBDs [20]. Furthermore, DP limits neutrophil chemotaxis by inhibiting IL-8 secretion. Schmidt et al. demonstrated that DP also inhibits IL-8 release from human keratinocytes, which is induced by the binding of autoantibodies to the BP-180 antigen in BP patients [21].

In addition to its primary mechanisms of action on neutrophils, there is evidence in the literature that DP also affects other inflammatory mediators. Abe et al. demonstrated that high concentrations of DP reduce TNF-α levels in PBMCs of patients with cutaneous lupus erythematosus [22]. As TNF-α levels are elevated in both PV [23] and BP [24], this represents another potential mechanism for DP in treating n-eAIBDs, though further research is needed in these patient groups. Grando et al. found increased levels of prostaglandins and leukotrienes in the blister fluid of patients with BP and PV [25], and DP has been shown to reduce these inflammatory mediators, supporting its use in treating n-eAIBDs and its potential GCS-sparing effects [26,27]. Murthy et al.’s 2021 study demonstrated that DP effectively reduces inflammation in antibody transfer mouse models of MMP and BP-like EBA by decreasing neutrophil infiltration, leukotriene B4 production, and ROS formation [28]. While these findings cannot be directly extrapolated to humans, they corroborate previous studies indicating DP’s multifactorial role in mitigating inflammatory processes.

In our study, most patients with various types of n-eAIBDs responded positively to DP treatment, showing significant improvement and a reduction in GCS dosage. Although the drug’s side effects were generally manageable, two participants withdrew due to severe gastrointestinal symptoms. Despite including only patients with active disease, most achieved complete or partial remission after a year on minimal therapy. However, disease relapse rates increased over time.

Early reports from over 40 years ago highlighted DP as an effective adjuvant treatment for pemphigus diseases [29,30,31]. The only randomized placebo-controlled study, conducted by Werth et al. in 2008, confirmed DP’s effectiveness as a GCS-sparing agent in 19 patients with PV [32]. Unlike our study, some patients in Werth et al.’s study also received AZA or MMF with DP and GCSs, with DP doses reaching 200 mg/day. Approximately 80% of these patients reduced their GCS dose to 7.5 mg or less, consistent with our findings where about 80% of PV patients achieved remission or partial remission after a year. Our study used DP as monotherapy alongside GCSs in lower doses, suggesting that chronic low doses of DP (up to 50 mg/day) may be safer and more effective than higher doses. Almugairen et al. found that half of the patients who started on DP without GCSs later relapsed, underscoring DP’s role as an adjuvant and suggesting that increasing GCSs, rather than DP, is preferable when initial treatment fails [33]. In 2016, Baum et al. reported that DP at 50–150 mg/day significantly lowered GCS dosages in a group of 26 pemphigus patients, with 62% achieving complete or partial remission. However, 50% discontinued the treatment due to side effects. The researchers recommended starting with low DP doses and gradually increasing them. In our study, starting with 100 mg of DP and then reducing the dose resulted in milder side effects, supporting the strategy of maintaining low DP doses while adjusting GCSs based on the clinical condition [34]. Reports suggest that MTX may be effective for treating BP and PV [35]. However, Jain et al. found no significant difference in therapeutic effects between MTX combined with GCSs and GCSs alone in patients with mucosal or mucocutaneous PV [36]. Although no studies compare the effectiveness of DP and MTX, we propose that DP is preferable for treating n-eAIBDs with mucosal involvement, due to MTX’s side effects, such as mucocutaneous ulcerations.

Our results indicate that patients with PF achieved a significantly higher percentage of CRMT compared to those with PV. This is likely due to the milder nature of PF, allowing greater use of DP. Almugairen et al. noted that DP alone could be appropriate for mild PF cases [33]. Ishii et al. observed that PV patients required higher doses of GCSs than PF patients. They introduced DP only in the PF group, in combination with local or oral GCSs depending on disease severity. Despite the higher GCS and immunosuppressive drug doses in PV patients, the complete remission rate in the PF group was more than twice that in the PV group [37]. These findings are consistent with our study, where the CRMT in the PF group after 12 months was significantly higher than in the PV group. This supports the conclusion that PV is a more severe disease than PF but also demonstrates the effectiveness of DP in treating PF. In a 2009 literature review, Gurcan and Ahmed found that 14 out of 18 PF patients responded to DP treatment, with the remaining 4 achieving remission when a GCS was added [38]. In our study, the complete remission rate in the PF group was higher than after one year, as we initially administered high GCS doses, which were gradually reduced. When comparing our results with previous studies, it appears that lower initial doses of GCSs may be effective in PF patients, given the high efficacy of DP even as a monotherapy.

The 2022 EADV guidelines for the management of patients with BP classify the use of DP as controversial but permissible, particularly for patients with contraindications to GCSs and immunosuppressive drugs [39]. The 2021 EADV guidelines for patients with MMP recommend DP for treating mild forms of the disease and in combination with other drugs (GCSs, immunosuppressants) for more severe cases [40]. However, randomized controlled trials are still lacking for both conditions.

In our study, only four patients had BP, as this condition is typically managed with local GCSs, with oral GCSs used in more severe cases, and DP considered an adjuvant therapy. In 2009, Hoffman et al. collected data from 42 hospitals in Germany on the treatment of pemphigus and pemphigoid diseases, revealing that oral GCSs were the most common therapeutic method. AZA was the most frequently used first-line adjuvant drug, while DP was used as a first- and second-line adjuvant drug in 22% and 28% of medical centers, respectively [41]. The authors noted a faster therapeutic effect of DP compared to AZA and MMF. Further studies from Iran and Denmark reported the use of DP as an adjuvant therapy in only a few patients [42,43]. Although there are reports of the effectiveness of DP monotherapy in BP at doses of 100–200 mg/day, potentially combined with local GCSs [44,45], other studies have shown a very low effectiveness for such therapy [46,47].

In our cohort of BP patients, we introduced oral GCSs and utilized low doses of DP. This approach resulted in the highest dose reduction in GCSs and the highest disease remission rates after 6 months of DP therapy. However, after 12 months, GCS doses had to be increased, and two patients experienced disease relapse. In 2012, Tirado-Sánchez et al. studied 15 BP patients treated with oral prednisone, with 8 patients receiving AZA and 7 patients receiving DP (100 mg/day). All patients achieved disease remission within 6 weeks, regardless of the adjuvant drug, but no data on GCS dose reduction were provided [48]. In the first randomized controlled study published in 2017 by Sticherling et al., five BP patients treated with AZA and oral GCSs, and three patients treated with DP (1.5 mg/kg) and oral GCSs demonstrated a GCS-sparing effect, more pronounced with DP. Despite the small sample size, the authors suggested DP as the first-line adjunctive treatment in moderate and severe BP to minimize the GCS dosage [49]. In our group, four patients successfully reduced their GCS doses using very low doses of DP, although two patients experienced lesion recurrence. This may have been due to the low DP dose or the advanced average age of our patients (71.25 years), as DP efficacy is noted to be better in patients under 60 years of age, according to Piamphongsant and Pearson and Roberts [44,46].

In our cohort of nine patients with MMP, over half achieved CRMT by the end of the observation period, with one R. PRMT and R rates at 2 and 6 months were higher, indicating that prolonged DP therapy may enhance the treatment response. Regardless of treatment response, patients significantly reduced their oral GCS doses. Gurcan and Ahmed’s literature review on 202 MMP patients demonstrated an 84% clinical improvement with DP doses ranging from 25 to 200 mg, either as a monotherapy or in combination with other immunosuppressants or GCSs. Despite the broad DP dose range and unspecified observation periods, DP’s efficacy in MMP treatment is evident [38]. In 2012, Staines and Hampton reported remission in six MMP patients treated with MMF, DP (25–50 mg/day), and oral GCSs over 18 months, with reduced GCS and MMF doses [50]. After 18 months of follow-up, all achieved remission with a reduction in the GCS and MMF dose. In another study conducted by Johnson KB et al., 20 patients with the ocular form of MMP were treated with various immunosuppressive drugs, and a GCS was used only ad hoc and short-term. In their conclusions, the authors emphasized the significant effectiveness of MMF over other treatment methods and also emphasized the low effectiveness of DP (only 25% of patients achieved disease remission) [51]. Comparatively, in our study, one patient experienced relapse, and three achieved PRMT. These results suggest that DP’s efficacy varies by MMP form and that combining DP with other immunosuppressants may enhance outcomes. Our use of low-dose DP and GCSs alone resulted in less than half achieving full remission after one year, with most remaining in PRMT and R for the first six months.

In our cohort, side effects of DP were transient and manageable with symptomatic treatment. However, two patients discontinued DP due to severe gastrointestinal symptoms. Our observations indicate that low doses of DP generally do not pose significant health threats. Nevertheless, serious side effects such as hepatotoxicity [52], hypersensitivity [53], and hematologic complications including methemoglobinemia and agranulocytosis can occur [54]. To mitigate these risks, it is advisable to regularly monitor patients’ blood tests and methemoglobin levels for early detection of these conditions. Additionally, even mild side effects should be taken seriously as they may precede DP hypersensitivity syndrome [55].

The side effects of DP are linked to its liver-produced metabolite, dapsone hydroxylamine (DHX). DHX reacts with oxyhemoglobin to form methemoglobin, leading to various symptoms of methemoglobinemia, such as nausea, headaches, fatigue, shortness of breath, or even coma. In cases of methemoglobinemia, the amount of circulating DHX is crucial, indicating that side effects are dose-dependent [56]. Additionally, DHX is suspected to be toxic to bone marrow cells, potentially causing agranulocytosis, though further research is needed to determine the exact cause [57]. DHX has also been implicated in dapsone hypersensitivity syndrome through the formation of haptens and the secretion of antibodies against dapsone [58]. Furthermore, studies by Liu et al. and Zhang et al. have suggested that the HLA-B*13:01 allele may be associated with a higher incidence of DHS, but additional research is necessary to confirm this link [59,60].

Our study has certain limitations due to the small sample size, which is attributable to the rarity of n-eAIBDs and the exclusion criteria applied. The limited availability of DP in Poland also contributed to the reduced study cohort. Additionally, the study’s reliance on patient descriptions and photographic documentation represents another limitation. Extending the follow-up period beyond one year could enhance a study’s robustness. Although our study cohort lacked homogeneity, DP appears to be variably effective across different types of n-eAIBDs. Our study has demonstrated its impact on GCS reduction and the treatment response in each subgroup classified by n-eAIBD type.

## 5. Conclusions

The GCS-sparing effect of DP in treating n-eAIBDs arises due to its multifactorial impact on inflammation. DP inhibits neutrophil chemotaxis and ROS formation and reduces the secretion of prostaglandins, leukotrienes, and various interleukins. Current data suggest that early inclusion of DP in n-eAIBD treatment is beneficial, as it targets the initial inflammatory processes. However, further research is needed to precisely understand DP’s impact on inflammatory mediator concentrations and to determine the optimal dose for maximal anti-inflammatory efficacy.

DP is a valuable medication in the treatment of n-eAIBDs and can be effectively used in conjunction with GCSs, followed by a subsequent dose reduction. At low doses, up to 50 mg/day, DP exhibits a favorable safety profile with mild side effects and possesses immunomodulatory properties. DP should be considered an adjunctive therapy, and in cases where there is no therapeutic response, more aggressive treatment approaches should be adopted. Given the adverse effects associated with GCSs, DP is especially beneficial for elderly patients and those with contraindications to GCSs.

Among pemphigus diseases, DP is particularly effective in PF, significantly reducing GCS doses and achieving complete remission. In PV, DP also exhibits a GCS-sparing effect; however, long-term management often requires other immunosuppressive treatments.

In the BP group, DP is an underrated adjuvant with GCS-sparing properties and fewer side effects compared to AZA. For MMP, DP effectively reduces GCS doses but should not be used as a monotherapy. Combining DP with other immunosuppressants, particularly MMF, appears promising.

From a financial perspective, DP is a cost-effective alternative compared to IVIG and RTX, offering a significant advantage in low-income settings. Our observations suggest that integrating DP into GCS therapy is worthwhile for patients where RTX and IVIG are either contraindicated or impractical due to logistical constraints. Furthermore, DP can be used with other immunosuppressive medications, allowing patients to extend the time to obtain other forms of therapy and enhancing the therapy’s effectiveness.

Further research is necessary to identify which patient populations will derive the greatest benefit from DP treatment and to compare its efficacy with other n-eAIBD therapies. It is important to tailor the DP dosage to the patient’s clinical condition and to establish factors that influence the drug’s dosage and treatment duration, along with guidelines for dose reduction and maintenance therapy.

## Figures and Tables

**Table 1:** Demographic data divided by type of n-eAIBDs diagnosis.

Disease Type	Number of Patients	Number of Women (Percentage)	Age (Years)	Baseline Prednisone Equivalent Dosage (mg)
PV	19	63.16	55.11 ± 12.76	26.11 ± 12.97
PF	9	55.56	60.89 ± 10.83	24.29 ± 14.74
MMP	9	44.44	73.89 ± 8.43	22.5 ± 4.33
BP	4	25	71.25 ± 4.44	23.75 ± 9.92

PF, pemphigus foliaceus; PV, pemphigus vulgaris; MMP, mucous membrane pemphigoid; BP, bullous pemphigoid.

**Table 2:** Occurrence of concomitant diseases (percentage) divided by type of n-eAIBDs diagnosis.

Disease Type	Number of Patients	AH	T2DM	HF	COPD	HT
PV	19	26.32	15.79	5.26	5.26	31.58
PF	9	33.33	22.22	0	11.11	33.33
MMP	9	33.33	33.33	22.22	22.22	22.22
BP	4	75	50	25	25	25

PF, pemphigus foliaceus; PV, pemphigus vulgaris; MMP, mucous membrane pemphigoid; BP, bullous pemphigoid; AH, arterial hypertension; T2DM, type 2 diabetes mellitus; HF, heart failure; COPD, chronic obstructive pulmonary disease; HT, hypothyroidism.

## Data Availability

The datasets generated and/or analyzed in the current study are not publicly available due to data privacy but are available from the corresponding author on reasonable request.
